# Rapid conversion of EUCOMM/KOMP-CSD alleles in mouse embryos using a cell-permeable Cre recombinase

**DOI:** 10.1007/s11248-013-9764-x

**Published:** 2013-11-07

**Authors:** Edward Ryder, Brendan Doe, Diane Gleeson, Richard Houghton, Priya Dalvi, Evelyn Grau, Bishoy Habib, Evelina Miklejewska, Stuart Newman, Debarati Sethi, Caroline Sinclair, Sapna Vyas, Hannah Wardle-Jones, Sanger Mouse Genetics Project, Joanna Bottomley, James Bussell, Antonella Galli, Jennifer Salisbury, Ramiro Ramirez-Solis

**Affiliations:** The Wellcome Trust Sanger Institute, Hinxton, Cambridgeshire, CB10 1SA UK

**Keywords:** Mouse, Genetics, Transgenics, Mammalian, Cre recombinase

## Abstract

We describe here use of a cell-permeable Cre to efficiently convert the EUCOMM/KOMP-CSD tm1a allele to the tm1b form in preimplantation mouse embryos in a high-throughput manner, consistent with the requirements of the International Mouse Phenotyping Consortium-affiliated NIH KOMP2 project. This method results in rapid allele conversion and minimizes the use of experimental animals when compared to conventional Cre transgenic mouse breeding, resulting in a significant reduction in costs and time with increased welfare benefits.

**Electronic supplementary material:**

The online version of this article (doi:10.1007/s11248-013-9764-x) contains supplementary material, which is available to authorized users.

## Introduction

The goal of the International Mouse Phenotyping Consortium (IMPC) (Brown and Moore 2012) is to generate knockout strains for all protein-coding genes in the mouse on a C57BL/6N genetic background, and to elucidate gene function by use of a broad spectrum high-throughput primary phenotyping screen. These phenotypes can then be studied in more depth by the scientific community at large with specialized areas of interest. To this end, the IMPC makes use of the knockout EUCOMM/KOMP-CSD embryonic stem cell (ESC) collection (Skarnes et al. 2011), which is derived from JM8 agouti or non-agouti C57BL/6N ES cells (Pettitt et al. 2009).

The EUCOMM/KOMP-CSD collection is based primarily around the ‘knockout-first’ (tm1a) allele which contains either an IRES:*lacZ* trapping cassette and a floxed promoter-driven *neo* cassette (promoter-driven) or an in-frame fusion of *lacZ* and *neo* with T2A peptide sequences included to allow expression of *lacZ* and *neo* as independent polypeptides (promoterless) inserted into an intron of the targeted gene. The mutagenic cassettes carry an *Engrailed* (En2) splice acceptor sequence and poly-A transcription termination signals which disrupt the targeted gene’s function while expressing the *lacZ* gene under the control of the endogenous promoter for studying gene expression.

**Fig. 1:**
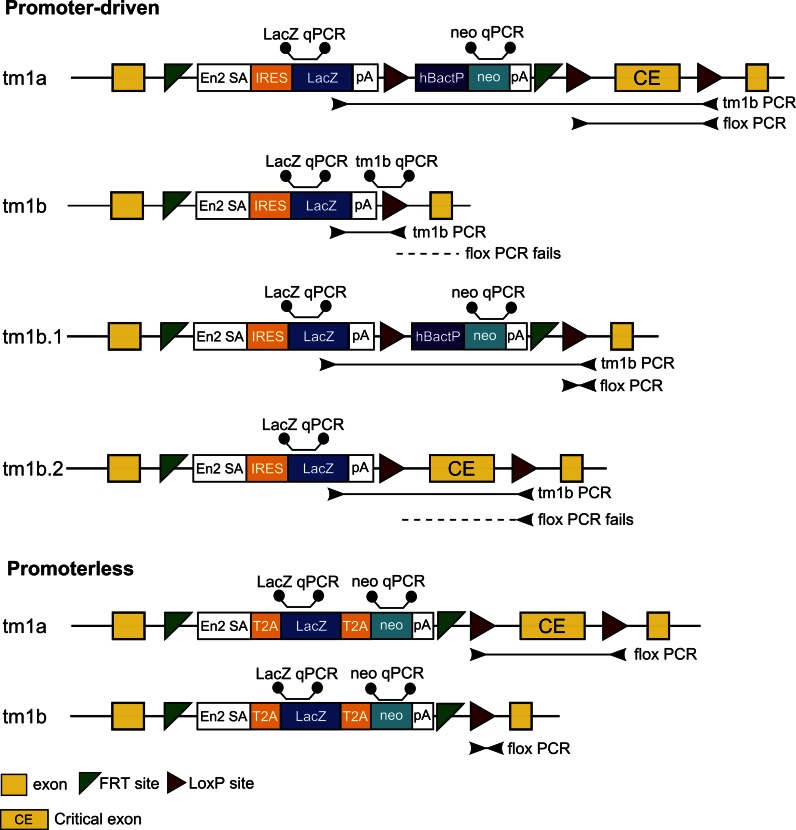
EUCOMM/KOMP-CSD allele structure and consequences of Cre-mediated excision. Mutant alleles based on the promoter-driven cassette have three loxP sites and can therefore have multiple outcomes after exposure to Cre recombinase. The desired result is tm1b where the *neo* selection cassette and floxed exon are removed. The tm1b.1 form removes only the critical exon and the tm1b.2 form removes only the *neo* selection marker, neither of which fulfills the requirement of the KOMP2 project. Alleles based on the promoterless cassette have only two loxP sites which flank the critical exon. Binding sites for the universal short range PCR and real-time qPCR genotyping assays are also shown

Exposure to Cre recombinase converts the tm1a to the tm1b allele to generate a non-conditional *lacZ*-tagged null allele without the critical exon, and removes the promoter-driven *neo* cassette if present (Fig. 1). The tm1a allele is the most versatile as in addition to the tm1b allele it can also be converted to a conditional mutant (tm1c) by exposure to FLP recombinase (Skarnes et al. 2011), and subsequent exposure to a tissue-specific Cre can allow the study of specific areas of interest. A recent review of the large resource of Cre drivers available for studying function in the mouse (Smedley et al. 2011) shows the versatility and robustness of the system in a myriad of tissues and developmental time points.

One of the goals of the Knockout Mouse Project phase 2 (KOMP2) is to produce tm1b strains to remove the *neomycin* promoter and critical exon from the tm1a allele in the mice produced; this is expected to alleviate potential off-target gene mis-regulation caused by the *neo* promoter (Pham et al. 1996; Scacheri et al. 2001; Ren et al. 2002; Meier et al. 2010) and to ensure that the allele is full null rather than a hypomorph (Shpargel et al. 2012). This requirement however has great implications on not only the extended time to convert the allele before colony expansion and generation of phenotyping cohorts, but also the considerable additional expense and number of animals used.

The established method for tm1a to tm1b conversion is to breed the mouse to a source of Cre expressed in the germ line (Schwenk et al. 1995); the Sanger Institute Mouse Genetics Project (Sanger MGP) (White et al. 2013) uses an X-linked CMV-Cre driver for this purpose (Su et al. 2002). After a two-generation breeding scheme, the offspring are genotyped to identify mice which have the target allele fully converted to tm1b but do not carry the Cre driver anymore, and these are used to establish and expand the colony for phenotyping.

The use of a cell permeable Cre to create recombination events in mouse embryos without the need for pronuclear injection has been described previously (Kim et al. 2009), using a His-TAT-NLS-tagged Cre (HTN-Cre) (Peitz et al. 2002). We present here a modified procedure for the use of HTN-Cre with EUCOMM/KOMP-CSD mouse strains in a high-throughput mouse production and IVF recovery pipeline, comparing the efficiency of recombination and time required for allele conversion with our existing CMV-Cre driver mouse breeding method.

## Methods

### Mouse production

The care and use of all mice in this study were in accordance with the UK Home Office regulations, UK Animals (Scientific Procedures) Act of 1986 and were approved by the Wellcome Trust Sanger Institute Ethical Review Committee.

### CMV-Cre breeding

The Sanger MGP and NIH KOMP2 projects require tm1a germplasm be archived for distribution to the wider scientific community, thus chimeras are mated with C57BL/6N wild type females. The Sanger MGP then archives sperm from tm1a strains for distribution to the scientific community via the EMMA (Wilkinson et al. 2010) and KOMP (Lloyd 2011) repositories. As part of the quality control procedures we perform in vitro fertilization (IVF) on an aliquot of frozen sperm and implant the resulting embryos into recipient females. The offspring are then genotyped to confirm the identity of the gene and that the archived sperm can successfully produce mutant animals. Heterozygous tm1a mutants are then mated to mice carrying the CMV-Cre allele with a preferred strategy of a tm1a heterozygote male paired with CMV-Cre homozygous females as the males can be cycled through multiple matings. Mutant F1 offspring are then backcrossed to wild type C57BL/6 N (Taconic) and the F2 progeny genotyped to detect full conversion of the allele to the tm1b form and the presence/absence of the CMV-Cre transgene (Supplementary Figure S1). Mice with the desired genotype (tm1b heterozygote, Cre transgene absent) are then used to expand the colony for phenotyping studies.

### Sperm cryo-preservation and recovery for in vitro fertilization (IVF)

Pooled sperm from two F1 males was cryopreserved in modified cryo-protective agent containing 18 % Raffinose, 3 % skimmed milk and 477 μM Monothioglycerol (mCPA) in cryo straws (Ostermeier et al. 2008) and held in liquid nitrogen storage tanks until required. In preparation for IVF, sperm pre-incubation dishes of TYH media + Methyl-β-cyclodextrin (MBCD) were set up and incubated at 37 °C (Takeo et al. 2008), and IVF fertilization dishes containing human tubal fluid (HTF) and reduced glutathione (GSH) were prepared (Takeo and Nakagata 2011). Sperm samples were thawed at 37 °C and the sperm was added to the pre-incubation TYH + MBCD drop for 30–40 min at 37 °C 5 % CO_2_ in air.

### Generation of oocytes and IVF

Three 4–5 week old C57BL/6NTac females per frozen sperm sample were super-ovulated by intraperitoneal (IP) injection of 5 IU of pregnant mare’s serum (PMSG) at 17:00 h (on a 12 h light/dark cycle, on at 07:00/off at 19:00) followed 48 h later by an IP injection of 5 IU human chorionic gonadotrophin (hCG). Oviducts were dissected at approximately 07:50 am on the day of the IVF, and cumulus-oocyte complexes were transferred into the IVF fertilization dish containing HTF + GSH. An aliquot of 20 μl of sperm from the pre-incubation dish was then added to the fertilization dish. After allowing 3–4 h for fertilization to occur the embryos were washed and cultured overnight in HTF at 37 °C, 5 % CO_2_ in air.

### HTN-Cre treatment of 2-cell embryos

HTN-Cre was sourced from Excellgen (product code RP-7) and stored at −20 °C. Following overnight culture 2-cell embryos were counted and put into a fresh drop of HTF media. A dish for HTN-Cre treatment was prepared as a series of five drops (Supplementary information S2) on the morning of the treatment. We found that it is important not to set up drops of HTN-Cre the night before as this seems to degrade the enzyme activity with resulting failure in excision (data not shown).

A total volume of 500 μl of DMEM + HTN-Cre solution in a 1 ml tube was prepared by adding 3 μl of HTN-Cre to 497 μl of DMEM (0.3 μM final concentration). Previous experiments using higher concentrations of HTN-Cre resulted in cell death in both 1 and 2-cell stage pre-implantation embryos (data not shown).

The 2-cell embryos were added sequentially to drops 1 and 2 to wash the embryos and remove bovine serum albumin (BSA) which may affect the HTN-Cre activity. The 2-cell embryos were then left in the HTN-Cre drop for 30–40 min for penetration of the cell membrane and Cre excision to take place. Longer exposure times resulted in developmental arrest and some cell lysis (data not shown). After this time, the Cre drop was flooded with HTF containing BSA to allow easier manipulation of the embryos. Embryos were then washed sequentially in HTF drops before being returned to the incubator prior to embryo transfer into a 0.5 days post coitum pseudo-pregnant female recipient produced by overnight mating to a vasectomised male (Nagy et al. 2003).

### HTN-Cre treatment of 4–8 cell cryo-preserved embryos

Conversion to tm1b via HTN-Cre was also performed using stocks of cryopreserved 4–8 cell preimplantation embryos. Methods for cryopreservation and thawing followed established controlled rate freezing methods (Renard and Babinet 1984). After the thawing procedure was completed the embryos were processed in the same way as for the IVF derived embryos with a 30 min culture in HTN-Cre followed by embryo transfer.

### HTN-Cre treatment of 2-cell Crlf3^tm1a(KOMP)Wtsi^ knockout embryos harvested from super-ovulated females

C57BL/6NTac females at 4–5 weeks of age were super-ovulated by intraperitoneal (IP) injection of 5 IU of pregnant mare’s serum (PMSG) at 13:00 h (on a 12 h light/dark cycle, on at 07:00/off at 19:00) followed 48 h later by an IP injection of 5 IU human chorionic gonadotrophin (hCG) and mated overnight to stud males carrying the knockout-first allele *Crlf3* ^*tm1a(KOMP)Wtsi*^. Females were checked the next morning for the presence of a copulation plug (0.5 days post coital).

At 1.5 d.p.c females were culled and the oviducts flushed for retrieval of 2-cell embryos. These were cultured in potassium simplex optimization media (KSOM) before being taken through the HTN-Cre treatment followed by embryo transfer as described for the IVF.

### Genotyping and tm1b conversion detection

A system of six universal assays (two end-point PCR and 4 copy-number qPCR) was developed to rapidly screen for full (tm1b) or partial (tm1b.1 or tm1b.2) conversion of the tm1a allele independently of the targeted gene (Supplementary information S3) and thus it is suitable for high-throughput systems and automation. This system was used to isolate the mice with the desired allele combinations for further breeding. The qPCR-based assays detect the presence and copy number of the Cre driver, *neo,* and *lacZ* cassettes, and conversion to tm1b in promoter-driven lines. The end-point assays amplify the floxed region of the mutant allele and detect the tm1b allele. The latter assays rely on a specific size of product in order to confirm the type of allele conversion and are therefore not suitable for a qPCR-based design. Interpretation of the results and how to identify the genotypes are shown in Fig. 1, Table 1 and Supplementary information S4.

**Table 1:** A combination of six PCR-based assays can be used to detect the Cre driver, the mouse genotype and any conversion from the tm1a to the tm1b forms of the mutant allele

Conversion	srPCR assay		qPCR assay		
	tm1b	Flox	tm1b_prom	LacZ count	*Neo* count
*Promoter*-*driven lines*					
No conversion (tm1a)	2,337 bp + CE size	Dependent on CE size	Fail	No change	No change
tm1b.1—only critical exon (CE) removed	2,291 bp	128 bp	Fail	No change	No change
tm1b.2—only *neo* removed	426 bp + CE size	Fail	Fail	No change	−1 copy
Full conversion to tm1b	380 bp	Fail	Pass	No change	−1 copy
*Promoterless lines*					
No conversion (tm1a)	1,580 bp + CE size	Dependent on CE size	Fail	No change	No change
tm1b	1457 bp	128 bp	Fail	No change	No change

Using this method, the mice can be genotyped in a mutant strain-independent manner using the LacZ qPCR assay which counts the number of copies of the cassette present and is unaffected by tm1b conversion. If no qPCR technologies are available then the short range PCR (srPCR) can be used to detect conversion and the mice genotyped by other methods (e.g. gene specific PCR assays designed to discriminate between the mutant and WT alleles). The tests show some redundancy and are used to cross-verify and interpret the results

Mice which show full conversion to tm1b are then re-genotyped by gene-specific methods using a custom TaqMan assay (Life Technologies) designed to detect the number of wild type copies of the targeted locus (Valenzuela et al. 2003); this confirms the gene identity and verifies the targeting.

### X-gal staining of a lacZ reporter line

To verify the activity of the cell permeable Cre, frozen sperm from the Rosa26-*loxP*-stop-*loxP*-*lacZ* reporter line Gt(ROSA)26Sor^tm1Sor^ (Soriano 1999) was used for IVF followed by treatment with or without HTN-Cre. The embryos were re-implanted and expression of *lacZ* was visualized on E10.5 embryos by staining with X-gal as described previously (Adams and Gale 2006), but with an overnight incubation at 4 °C.

## Results

### Conversion of the tm1a allele using CMV-Cre

**Fig. 2:**
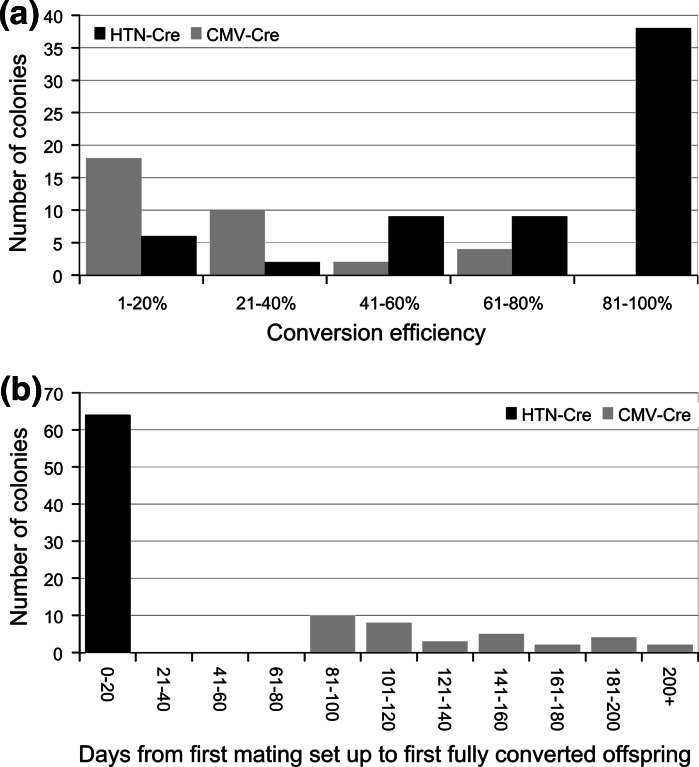
**a** Efficiency of conversion in HTN-Cre is much higher than if using CMV-Cre transgenic animals, with 27/64 strains showing 100 % conversion by genotyping. **b** The time taken to produce a mouse converted to tm1b with no Cre driver still present varies from 83 to over 200 days using CMV-Cre, whereas the treatment with HTN-Cre during IVF recovery takes less than 21 days

From a total of 51 strains that displayed excision by conventional breeding, 34 showed at least one F2 mouse with conversion to tm1b and absence of the Cre driver (Supplemental Table 1). An additional 7 strains showed conversion to tm1b or mosaic conversion including tm1b.1 and/or tm1b.2 with the Cre driver still present. The conversion rate, calculated as tm1b F2 mice detected/total F2 mutants, varied widely among mutant strains from 4.3 % in *Rbmx* up to 75 % in *Cdkn2aipnl.* The average frequency of conversion was only 21 % (191/926) of mutant mice from the F2 generation (and beyond if further back-crossing was required) showing conversion to tm1b and the absence of the Cre driver (Fig. 2a).

The number of generations required for allele conversion and the removal of the Cre transgene varied within and between genes depending on the initial and subsequent mating setup, and so total conversion time for the colony is shown instead. The time taken through breeding to reach conversion to tm1b and absence of the Cre driver ranged from 84 to 218 days (average = 131 days) from the date of the first tm1a x CMV-Cre mating set up (Fig. 2b). This delays the start of colony expansion and cohort breeding for phenotyping experiments by 16 weeks compared to phenotyping the tm1a allele. No correlation was detected between the size of the critical exon region and the conversion rate.

### Conversion of the tm1a allele using cell permeable HTN-Cre

Using the IVF recovery and subsequent HTN-Cre treatment, we saw at least one mouse with conversion to tm1b in 60/64 lines (Supplemental Table 2); the remaining four strains showed only mosaics of tm1b (which can still be useful if the conversion has also occurred in the germ line) and partial conversion to tm1b.1 and tm1b.2.

Compared to the CMV-Cre breeding method, the IVF recovery with HTN-Cre treatment takes just 20 days from the start of the procedure to the birth of fully converted offspring (Fig. 2b). Conversion rates were very high (Supplementary information S5) with 27 lines showing all F0 progeny converted to tm1b and an average of 79 % of the progeny across all 64 lines. This number compares favourably to the average of 21 % when using the CMV-Cre breeding method (Fig. 2a), and the 42 % reported on P14 pups by Kim et al. 2009 when using 3 μM HTN-Cre with a 1 h incubation. Results from both methods are summarized in Table 2. Again, no significant correlation was found between the conversion rate and the size of the floxed region.

**Table 2:** Summary of CMV-Cre transgenic breeding and cell permeable HTN-Cre

Method	Genes	Mutants genotyped	Conversion to tm1b and cre removed	% conversion	Average # days from initial mating to first tm1b mouse with cre removed
HTN-Cre—via IVF	64	423	333	79	20
CMV-Cre—natural mating	34	926	191	21	131

Conversion rate in HTN-Cre is nearly four times greater than using CMV-Cre transgenic breeding and the time needed for conversion is much faster

The F0 tm1b heterozygous mice recovered from IVF were used in expanding the colony by breeding them to wild type C57BL/6N. In a pilot study of 323 F1 mutant offspring from 29 different strains whose parents did not show any sign of mosaicism, 317 carried the correct form of the tm1b allele (98 %), showing that conversion occurred in the germ line as well as the soma of the F0 parents. The six remaining F1 mutants, which originated from 3 different colonies and had tm1b siblings, carried the incorrect form of the tm1b allele. This indicates that even when mosaicism is not detected somatically in the F0 ear sample used for genotyping, it can still be present at a very low frequency in the germline. These data highlight the need to confirm the conversion in the F1 generation before embarking on any larger breeding regimes.

### Conversion of 4–8 cell cryo-preserved embryos and 2-cell harvested embryos using HTN-Cre

Embryos cryogenically preserved from the EUCOMM/KOMP-CSD strain Prmt5^tm2a(EUCOMM)Wtsi^ were thawed and treated with 0.3 μM HTN-Cre using the same protocol as for the IVF recovery. Five mutant pups were detected, three of which converted to tm1b, with one mosaic and one showing no conversion. In a separate experiment, 10 mutant pups were recovered from *Crlf3* ^tm1a(KOMP)Wtsi^ 2-cell embryos harvested from super-ovulated wild type females mated to *Crlf3* ^tm1a(KOMP)Wtsi^ heterozygous males, 9 of which showed conversion to tm1b and 1 was mosaic for tm1a/b.

### Confirmation of HTN-Cre activity with a lacZ reporter line

**Fig. 3:**
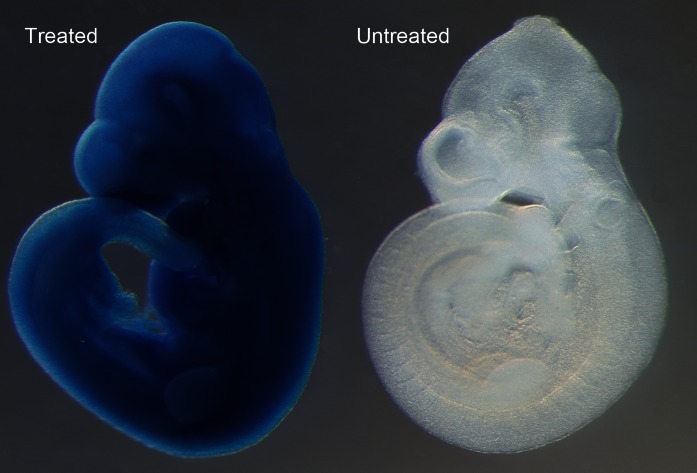
X-gal staining of a reporter line treated with cell permeable Cre results shows complete excision of the *neo* cassette, allowing ubiquitous expression of the *lacZ* gene

To confirm the level of activity of the cell permeable Cre we performed X-gal staining on an HTN-Cre treated Gt(ROSA)26Sor^tm1Sor^ *lacZ* reporter strain (Soriano 1999). Six out of seven samples treated with the cell permeable Cre showed ubiquitous expression of the *lacZ* gene in the embryo (Fig. 3), and complete excision of the *neo* cassette confirmed by a lack of amplification with a *neo*-specific assay when genotyping the yolk sacs. Conversely, the embryo which did not show any *lacZ* staining also did not show excision of the *neo* cassette. Untreated embryos showed no *lacZ* expression, as predicted.

## Discussion

Our results show that although CMV-Cre can be used to convert EUCOMM/KOMP-CSD tm1a knockout-first alleles to the tm1b non-conditional *lacZ*-tagged null allele, the time and resources necessary do not support its use as a viable long-term tool for the IMPC and KOMP2 projects.

The preferred configuration for the first step in the breeding strategy consists in crossing the heterozygous male carrying the tm1a allele to a homozygous female carrying the CMV-Cre driver. All the F1 progeny will be heterozygous or hemizygous for the X-linked Cre driver, but only half of the progeny will also carry the tm1a allele. As the CMV-Cre driver can act during the development of these F1 mice, somatic excision can be detected in ear samples. However, the detection of somatic excision does not guarantee excision in the germ line, and vice versa, the lack of detectable somatic excision does not preclude germ line excision. For example, in the *Dbn1* strain, all 9 F1 double heterozygotes showed complete somatic conversion to tm1b allele, yet when those mice were bred to wild type, only 1/11 F2 progeny carried the tm1b allele. Alternatively, in the *3110035E14Rik* line no somatic conversion at all was detected in the F1, yet when bred to wild type, 8/28 F2 progeny carried the tm1b allele. For this reason only F2 mice carrying the tm1b excised allele and no Cre driver transgene were assumed to be non-mosaic, as we could not determine whether the conversion happened in the germ line of the parent or was happening somatically in the offspring with the possibility of not transmitting to the next generation.

Since our project was initiated, other drivers have become available which may be more suitable for high-throughput projects, for example the Gt(ROSA)26Sor^tm1(ACTB−cre,−EGFP)Ics^ allele (Birling et al. 2011) which has the advantage of maternal expression for enhanced recombination efficiency and a fluorescent eGFP marker for ease of tracking the allele. The stipulation of the IMPC that all mice should be produced in a pure C57BL/6N background precludes some other available drivers such as the Stella Cre (Liu et al. 2011) even though they are reported as being highly efficient.

Alternative approaches for Cre-induced excision have focused on microinjection of either Cre mRNA (de Wit et al. 1998), or protein (Luckow et al. 2009) to produce recombination. Although reported as successful, these have the relative limitation of injection rates in a high-throughput project. The work by Kim et al. showed a potential avenue to avoid these drawbacks if it could be adapted to a high-throughput environment, which we demonstrate here in over 60 EUCOMM/KOMP-CSD strains processed and analysed.

Kim et al. 2009 reported that only approximately 40–49 % of embryos survived culture to blastocyst after treatment with 3 μM HTN-Cre for 1 h compared to an untreated control group. From a study of 17 experiments with 11 distinct EUCOMM/KOMP alleles, the Rosa26 *lacZ* reporter and C57BL/6N WT mice, we found that using 0.3 μM HTN-Cre increased the survival rate to blastocyst to an average of 94 % (Supplemental Table 3), ranging from 79 to 100 %. We also determined that 0.3 μM HTN-Cre treatment had no detrimental effect on the survival rates of the embryos transferred to pseudo-pregnant females, compared to those from routine IVFs without treatment (Table 3).

**Table 3:** HTN-Cre treatment has no detrimental effect on the survival of the embryos during transfer to pseudo-pregnant females and subsequent births

HTN-Cre treated	# Recipients	# non-pregnancies	# Embryos transferred	# Pups born	% Pups born
Yes	177	27 (15 %)	2,318	975	42
No	360	83 (23 %)	4,976	1,800	36

Perhaps the most striking difference however was in pup survival rates. Kim et al. reported that when using 3 μM HTN-Cre with a 1 h incubation a third of pups died within a day of being born (80 % of which showed excision of the *neo* cassette in the *lacZ* reporter line), and growth retardation in some of the survivors, strongly suggesting that the Cre was having a toxic effect on the developing embryo. By using 0.3 μM HTN-Cre for ~ 40 min we observed only 3/901 pups (0.33 %) from 66 colonies died within the first week of birth, raising the survival rate from 66 to 99.7 %. Efficiency of excision was also very high at 79 % in F0 mutants, compared to the 42 % originally reported in P14 pups by Kim et al.

One major advantage of the HTN-Cre protein method over the use of the CMV-Cre transgenic mouse strain is that breeding at least two generations, with its consequent increase in cage costs, is no longer required to obtain conversion and eliminate the Cre allele; and there is no need for a large core colony of Cre driver mice to be established and maintained. Additionally, a much higher proportion of treated mice carry the tm1b allele. Full conversion to tm1b, rather than mosaic and partial conversion to the tm1b.1 or tm1b.2 forms was detected in all but 16 % of mutant F0 animals that displayed any conversion, and the latter can be screened out easily at the F1 stage. Additionally, as IVF and QC of our cryopreserved sperm resource form part of our mouse production and archiving pipeline, the HTN-Cre mediated conversion could be added with little extra effort or expense. These advantages have large implications for cost and time savings and also a significant reduction in the numbers of animals needed for conversion detection and subsequent expansion breeding, in line with the Sanger Institute’s commitment to the 3Rs (Fenwick et al. 2011; NC3Rs 2012). As not all transgenic laboratories have access to IVF facilities, we have also shown that the HTN-Cre technique works well with both cryogenically preserved embryos and embryos derived from mating super-ovulated females to stud males. Although actual costs will vary between different transgenic centres, by using the HTN-Cre method all centres, independently of their mouse production throughput, should realize savings compared with the breeding strategy, mainly due to much lower cage/week expenses.

## Electronic supplementary material

Below is the link to the electronic supplementary material.

Supplementary material 1 (PDF 28 kb)

Supplementary material 2 (PDF 142 kb)
