# Supplementary material for: Rapid conversion of EUCOMM/KOMP-CSD alleles in mouse embryos using a cell-permeable Cre recombinase
Source: Transgenic Res. 2013 Nov 7;23(1):177–85. doi: 10.1007/s11248-013-9764-x (PMC3890051; doi:10.1007/s11248-013-9764-x)
Supplement: Supplementary file 1 — Supplementary material 1 (PDF 28 kb) [file 11248_2013_9764_MOESM1_ESM.pdf]

Supplemental Table 1. Rates of conversion to tm1b using CMV-Cre and natural matings.

Gene	Critical exon region (bp)	F1 mutants genotyped	F1 mutant mice showing somatic conversion	% F1 mice showing somatic conversion	F2 mutants genotyped	F2 mutants showing full tm1b conversion and no Cre allele present	% conversion to tm1b and no Cre allele present per strain	# days from first mating set up to first fully converted tm1b mouse detected with no Cre
2610318N02Rik	718	13	11	85%	30	8	27%	93
3110035E14Rik	590	5	0	0%	28	8	29%	178
Aldh3b1	3689	22	8	36%	4	1	25%	113
Ankrd6	0	5	2	40%	15	3	20%	141
Anxa9	1864	14	5	36%	19	2	11%	96
Ap2a2	744	11	5	45%	18	2	11%	91
Cdkn2aipnl	629	16	5	31%	4	3	75%	84
Cnbd1	595	33	10	30%	51	12	24%	95
Cyp2r1	758	24	9	38%	32	7	22%	94
Dbn1	2019	9	9	100%	11	1	9%	176
Dennd1c	1311	17	3	18%	48	4	8%	154
Exoc3l2	947	15	5	33%	20	7	35%	189
Fbxo33	2325	31	1	3%	6	1	17%	145
Gpr152	1975	20	13	65%	11	1	9%	127
Leprot	653	23	4	17%	94	9	10%	190
Lgals7	1357	6	2	33%	18	3	17%	137
Man2b2	699	16	13	81%	20	7	35%	104
Mybphl	2191	21	8	38%	83	4	5%	145
Prrg2	1545	12	7	58%	8	4	50%	115
Rbmx	760	28	2	7%	23	1	4%	131
Rhox13	567	31	21	68%	35	3	9%	218
Ropn1l	556	16	7	44%	36	16	44%	95
Selk	1106	29	4	14%	88	17	19%	103
Sfxn3	1730	8	4	50%	34	12	35%	99

Slitrk4	4501	19	18	95%	24	15	63%	115
Smpd4	964	11	1	9%	38	13	34%	144
Tatdn3	616	9	5	56%	5	1	20%	94
Tceal5	1345	23	18	78%	8	5	63%	191
Tmc3	689	9	3	33%	6	2	33%	102
Tomm20l	688	4	2	50%	11	1	9%	195
Trim29	3450	12	6	50%	24	4	17%	88
Wnt16	877	9	9	100%	8	5	63%	111
Zfp719	689	14	1	7%	35	3	9%	206
Zkscan17	646	22	5	23%	31	6	19%	103
**Total**		**557**	**226**	**41%**	**926**	**191**	**21%**	

Supplemental Table 2. Conversion rates to tm1b using cell permeable HTN-Cre via IVF

Gene	Critical exon region (bp)	Total hets	Tm1a	Tm1b	Mosaics detected	incorrect allele conversion	% full conversion
1700007K13Rik	555	14	0	11	2	1	79%
Adamts3	997	3	0	3	0	0	100%
Alox12e	3036	5	0	5	0	0	100%
Ankrd6	0	6	4	1	1	0	17%
Ap4e1	671	5	0	5	0	0	100%
Camkmt	843	13	0	13	0	0	100%
Casc4	671	3	0	2	1	0	67%
Ccdc127	722	12	0	5	3	4	42%
Cmbl	822	4	1	3	0	0	75%
Cpt2	647	14	0	13	1	0	93%
Ctr9	1734	5	2	0	3	0	0%
Cttnbp2	2234	14	0	13	1	0	93%
D630023F18Rik	979	8	0	6	1	1	75%
Dhodh	643	5	1	0	2	2	0%
Dip2a	1576	8	3	4	1	0	50%
Dnajc8	532	3	0	3	0	0	100%
Dnase1l2	0	7	0	7	0	0	100%
Eci3	613	9	0	9	0	0	100%
Ell2	659	5	2	3	0	0	60%
Exoc3l2	947	5	0	2	1	2	40%
Fbxo33	2325	1	0	1	0	0	100%
Fbxo7	659	5	0	3	2	0	60%
Galnt18	708	9	0	9	0	0	100%
Gm13125	979	2	0	1	1	0	50%
Gm16515	682	2	0	2	0	0	100%
Gmnc	1643	13	0	12	0	1	92%
Hibadh	721	6	0	6	0	0	100%

Kif24	802	5	0	5	0	0	100%
Kif3b	2086	9	0	8	1	0	89%
Klf17	1606	2	0	1	0	1	50%
Krt7	756	13	0	11	2	0	85%
Lonrf3	845	4	0	4	0	0	100%
Medag	612	9	0	9	0	0	100%
Metrnl	953	9	0	8	1	0	89%
Mroh4	1321	14	1	9	4	0	64%
Mrps5	1887	3	0	3	0	0	100%
Nubpl	698	6	0	6	0	0	100%
Nxn	701	4	1	3	0	0	75%
Pced1a	1477	2	0	1	0	1	50%
Pdcd2	873	10	0	10	0	0	100%
Pdia4	926	6	1	1	4	0	17%
Plscr2	1144	11	0	8	3	0	73%
Ppil3	649	17	0	16	1	0	94%
Prkab1	728	13	2	11	0	0	85%
Rbmx	760	5	1	4	0	0	80%
Reg3d	960	11	0	9	2	0	82%
Rnf157	815	3	0	3	0	0	100%
Rundc1	524	3	0	3	0	0	100%
Rwdd1	668	3	1	0	2	0	0%
Sgsm1	751	6	0	5	1	0	83%
Slc25a28	763	10	0	10	0	0	100%
Smpd4	964	6	3	0	3	0	0%
Stard8	1974	6	0	5	1	0	83%
Tgfb1i1	2052	6	0	6	0	0	100%
Tmc3	689	4	1	2	1	0	50%
Trappc10	1162	5	0	5	0	0	100%
Trmt2a	1596	3	0	3	0	0	100%

Vps13a	631	3	0	3	0	0	100%
Zfp182	1029	7	0	3	4	0	43%
Zfp287	524	3	1	1	0	1	33%
Zfp408	3302	3	0	2	1	0	67%
Zfp616	1193	5	0	5	0	0	100%
Zfp84	2795	3	0	3	0	0	100%
Zfyve28	732	5	0	5	0	0	100%
**Total**		**423**	**25**	**333**	**51**	**14**	**79%**

Supplemental Table 3. Embryos cultured from 2-cell to blastocysts and survival rates.

Gene	# Embryos Cultured	# Survived to blastocyst	% Survival
Adamts3	10	10	100
Alox12e	17	14	82
WT C57BL/6N (Taconic)	14	13	93
Dip2a	17	16	94
Klf17	19	19	100
Klf17	12	11	92
Rosa26 lacZ reporter	19	19	100
Rosa26 lacZ reporter	11	10	91
Mkrn2	7	7	100
Mrps5	10	9	90
Pced1a	28	26	93
Pld3	14	11	79
Prkab1	22	20	91
Rundc1	17	15	88
Rundc1	13	13	100
Stard8	20	20	100
Tmc3	16	16	100
**Total**	**266**	**249**	**94**
