# Supplementary material for: Rapid conversion of EUCOMM/KOMP-CSD alleles in mouse embryos using a cell-permeable Cre recombinase
Source: Transgenic Res. 2013 Nov 7;23(1):177–85. doi: 10.1007/s11248-013-9764-x (PMC3890051; doi:10.1007/s11248-013-9764-x)
Supplement: Supplementary file 2 — Supplementary material 2 (PDF 142 kb) [file 11248_2013_9764_MOESM2_ESM.pdf]

Supplementary information

S1 - CMV-Cre takes a minimum of two generations to convert tm1a to tm1b with the Cre driver removed. The ratio of fully converted mice to other genotypes is also very low, even if the conversion rate to tm1b is 100%.

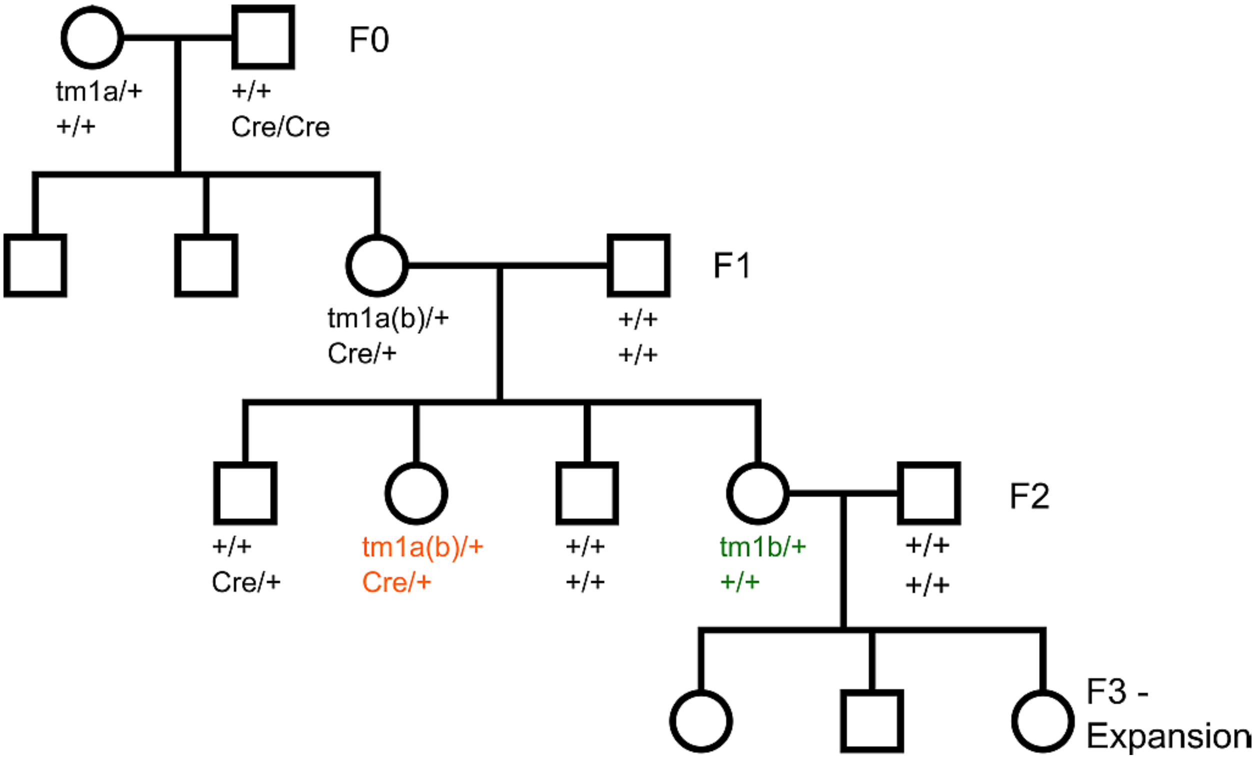

S2 - Cell permeable Cre incubation 2 cell embryos. Drops 1 and 2: 2 x 130µl DMEM wash drops. Drop 3: 1 x 50µl DMEM + HTN-Cre. Drops 4 and 5: 2 x 130µl HTF wash drops post Cre drops 4 and 5. All drops are overlaid with mineral oil.

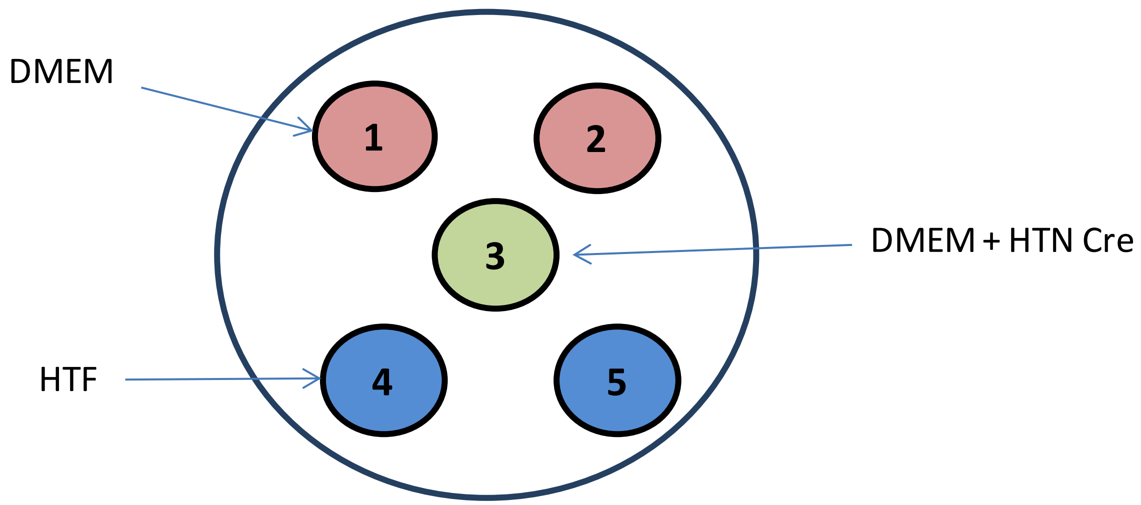

S3 – PCR conditions

Short range primer sequences and PCR assays for tm1b and Cre detection

Primer name	Sequence
Cre_F	CATTTGGGCCAGCTAAACAT
Cre_R	TAAGCAATCCCCAGAAATGC
Floxed PNF	ATCCGGGGGTACCGCGTCGAG
Floxed LR	ACTGATGGCGAGCTCAGACC
Tm1b_prom_F	CGGTCGCTACCATTACCAGT

Assay	F primer	R primer	Size (bp)	Type
Cre	Cre_F	Cre_R	233	Cre
Flox	Floxed PNF	Floxed LR	variable	LoxP
Tm1b	Tm1b_prom_F	Floxed LR	380	cassette

Short Range PCR reagents and cycling conditions

Reagent	volume (ul)
Primer 1 (10uM)	0.3
Primer 2 (10uM)	0.3
MgCl2 (50mM)	0.45
10x Buffer	1.5
dNTP (100mM)	0.15
Taq*	0.15
ddH_2O	11.15
DNA	1
**Total**	**15**

*Platinum Taq is used in the PCR reaction (Invitrogen)

PCR conditions

1 94 ^{o}C 5 min
2 94 ^{o}C 30 sec
3 58 ^{o}C 30 sec
4 72 ^{o}C 45 sec
5 Go to '2' + 34 cycles
6 72 ^{o}C 5 min
7 12 ^{o}C forever

qPCR assay sequences

Assay	F primer	R primer	Probe
LacZ	GGAGTGCGATCTTCCTGAGG	CGCATCGTAACCGTGCATC	CGATACTGTCGTCGTCCCCTCAAACTG
Neo	GGTGGAGAGGCTATTCGGC	GAACACGGCGGCATCAG	TGGGCACAACAGACAATCGGCTG
Tm1b_prom	GTCCAAACTCATCAATGTATCTTATCATGT	GATGGCGAGCTCAGACCATAA	TGGATCCGGAATAACTTCGTA
Cre	ACGTACTGACGGTGGGAGAA	GTGCTAACCAGCGTTTTCGTT	CTGCCAATATGGATTAACA

qPCR reagents and cycling conditions

Reagent	Volume (ul)
2x GTXpress buffer*	5
60x TaqMan assay	0.166
ddH_2O	3.334
20x endogenous probe (Tfrc)*	0.5
DNA	1

*Applied Biosystems

95ºC 20sec } x1

95ºC 10sec
60ºC 30sec } x35

S4 – Genotyping and tm1b conversion detection using the universal short range PCR assays

*S*ize differences in the end-point PCR reactions can detect partial and mosaic conversion of the mutant allele (data shown for promoter-driven lines). In this example the tm1b assay detects full conversion to tm1b in three samples and partial conversion to tm1b.2 in two others. The flox assay (performed on different samples and shown here for illustrative purposes) detects conversion to tm1b.1 in two samples and no conversion in two others. The remaining sample shows mosiacism where both the tm1a and tmb.1 forms are detected.

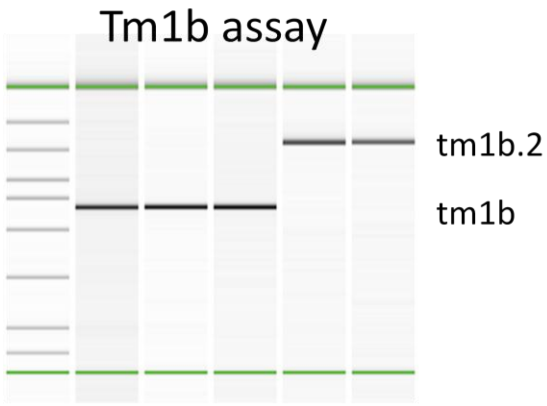

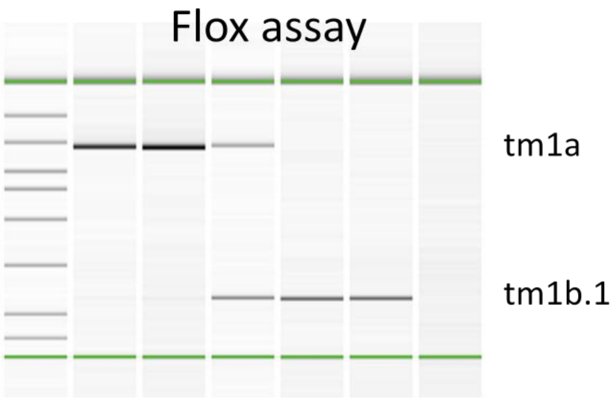

S5 – Genotyping of cell permeable Cre-treated samples

All nine samples which are heterozygous for LacZ show tm1b conversion. Mouse MZBG3.1a also shows some mosaic conversion to tm1b.1. No tm1a is detected in any of the samples. The upper and lower green bands are alignment markers used by the fragment analysis software (Qiagen)

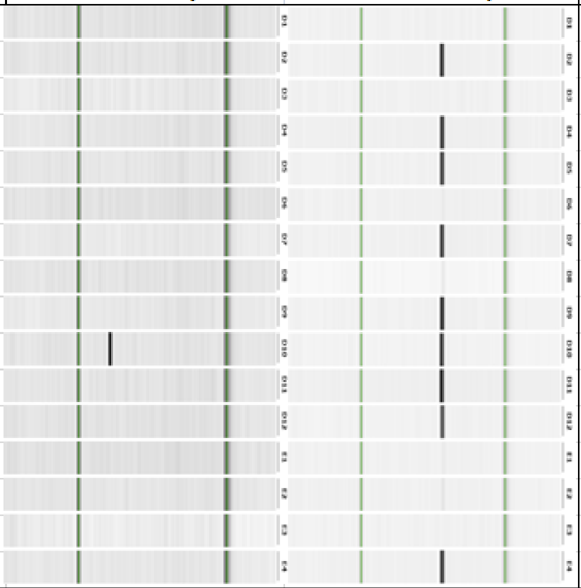

	SR result		qPCR results			Genotypes	
Mouse	Flox assay	Tm1b assay	LacZ	Neo	Tm1b_prom	Tm1(a)	Tm1(b)
MZBG1.1a			+/+	+/+	+/+	+/+	+/+
MZBG1.1b			+/-	+/+	+/-	+/+	+/-
MZBG1.1c			+/+	+/+	+/+	+/+	+/+
MZBG1.1d			+/-	+/+	+/-	+/+	+/-
MZBG1.1e			+/-	+/+	+/-	+/+	+/-
MZBG2.1a			+/+	+/+	+/+	+/+	+/+
MZBG2.1b			+/-	+/+	+/-	+/+	+/-
MZBG2.1c			+/+	+/+	+/+	+/+	+/+
MZBG2.1d			+/-	+/+	+/-	+/+	+/-
MZBG3.1a			+/-	+/+	+/-	+/+	+/-
MZBG3.1b			+/-	+/+	+/-	+/+	+/-
MZBG3.1c			+/-	+/+	+/-	+/+	+/-
MZBG3.1d			+/+	+/+	+/+	+/+	+/+
MZBG3.1e			+/+	+/+	+/+	+/+	+/+
MZBG3.1f			+/+	+/+	+/+	+/+	+/+
MZBG3.1g			+/-	+/+	+/-	+/+	+/-
